# Off the deep end: What can deep learning do for the gene expression field?

**DOI:** 10.1016/j.jbc.2022.102760

**Published:** 2022-11-30

**Authors:** Ana-Maria Raicu, Justin C. Fay, Nicolas Rohner, Julia Zeitlinger, David N. Arnosti

**Affiliations:** 1 Cell and Molecular Biology Program, Michigan State University, East Lansing, Michigan, USA; 2 Department of Biology, University of Rochester, Rochester, New York, USA; 3 Stowers Institute for Medical Research, Kansas City, Missouri, USA; 4 Department of Molecular & Integrative Physiology, University of Kansas Medical Center, Kansas City, Kansas, USA; 5 Department of Pathology & Laboratory Medicine, University of Kansas Medical Center, Kansas City, Kansas, USA; 6 Biochemistry and Molecular Biology Program, Michigan State University, East Lansing, Michigan, USA

**Keywords:** deep learning, gene expression, transcription

## Abstract

After a COVID-related hiatus, the fifth biennial symposium on **Evolution and Core Processes in Gene Regulation** met at the Stowers Institute in Kansas City, Missouri July 21 to 24, 2022. This symposium, sponsored by the American Society for Biochemistry and Molecular Biology (ASBMB), featured experts in gene regulation and evolutionary biology. Topic areas covered enhancer evolution, the *cis*-regulatory code, and regulatory variation, with an overall focus on bringing the power of deep learning (DL) to decipher DNA sequence information. DL is a machine learning method that uses neural networks to learn complex rules that make predictions about diverse types of data. When DL models are trained to predict genomic data from DNA sequence information, their high prediction accuracy allows the identification of impactful genetic variants within and across species. In addition, the learned sequence rules can be extracted from the model and provide important clues about the mechanistic underpinnings of the *cis*-regulatory code.

## Interpreting the *cis*-regulatory sequence rules to obtain a mechanistic understanding of gene regulation

A sought-after goal of the gene regulation field is to decode enhancer grammar (1). How do transcription factor (TF) binding motifs within an enhancer combine to generate its unique activity? Can we learn the enhancer grammar to create synthetic spatially or temporally regulated enhancers? The great advantage of deep learning (DL) models over traditional methods is that they learn complex *cis*-regulatory rules in a precise and unbiased manner, allowing for new sequence rules to be discovered. Identifying these rules is done after model training and is not trivial; yet, a variety of interpretation tools already exist to obtain the important sequence features and their rules of interactions. In this manner, DL models reliably reveal the binding motifs of TFs and provide important clues as to how the motifs combine to produce an experimental outcome.

Interpreting DL models can therefore reveal novel mechanistic insights that can then be tested experimentally. For example, **Alexander Stark** (IMP, Vienna) discussed his laboratory’s recent application of DL to their STARR-seq method to predict enhancer activity from DNA sequence (2). DeepSTARR can be successfully used to design synthetic enhancers with desired activities. **Julia Zeitlinger** (Stowers Institute) described her laboratory’s work on applying DL to understand the *cis*-regulatory rules of enhancers during *Drosophila* embryogenesis. Using BPNet (3) and chromBPNet as DL models, they uncovered TF cooperativity and sequences critical to opening chromatin, revealing a different effect for high *versus* low affinity motifs. **Shaun Mahony** (Pennsylvania State University) also explored the mechanistic basis of chromatin accessibility by studying the evolutionary impacts of *FOX* gene paralogs (4). Their DL approach specifically modeled chromatin state and DNA sequence to predict whether individual paralogs required prior chromatin accessibility for binding.

Several talks focused on further improving our ability to extract *cis*-regulatory information from DL models. **Sara Mostafavi** (University of Washington) talked about her approaches to identify sequence features important for determining chromatin states in diverse human immune cells. She discussed approaches to unlock elements in the DL algorithms that were informative and reproducible, including identifying the number of active motifs found at diverse enhancers (5). **Vivekanandan Ramalingam** (Kundaje laboratory; Stanford University) illustrated how the Kundaje laboratory extracts important DNA sequences from a DL model to identify the distance rules by which TF motifs cooperate in binding and opening chromatin (3). He also shared dynseq, a browser tool for visually exploring the sequences that were learned by a DL model (6). These examples highlight the importance of further tool development for motif identification and functional contributions to enhancer activity.

A strength of DL models is that they can learn sequence rules in an unbiased manner. Reassuringly, the learned rules can often be matched to known processes involved in gene regulation. For example, the learned TF binding motifs and their affinities correspond remarkably well to biophysical models of TF binding. Therefore, an important goal is to combine DL with biophysical models of transcription. Along this line, **Justin Kinney** (Cold Spring Harbor Laboratory) discussed Mave-NN, a computational framework for integration of diverse gene expression data to make DL accessible to a biological user base (7). These biophysical models will illuminate the functional properties of gene switches such as those studied using optogenetic technology by **Hernan Garcia** (UC Berkeley) in the *Drosophila* embryo (8).

## Placing extracted sequences into gene regulatory networks and developmental processes

Since sequence information plays a central role in the fields of gene regulation, development, and evolution, sequence-centered DL models are an excellent way to promote cross-fertilization between these fields, which was the overarching theme for this American Society for Biochemistry and Molecular Biology (ASBMB)-sponsored conference. Evolution is a crucial trove of knowledge for understanding gene regulation, and conversely, an understanding of gene regulation is a key to unlocking evolutionary processes. Deep learning can therefore have a significant impact toward accelerating this dialogue between fields. One path forward is to integrate the sequence information extracted from DL models with other data modalities to construct gene regulatory networks (GRNs). For example, to characterize key players in zebrafish inner ear regeneration, **Erin Jimenez** (Shawn Burgess laboratory; NIH) used single-cell ATAC-seq and RNA-seq to identify activated enhancers during regeneration. Using DL, she uncovered a role for the Sox and Six TFs in coordinately regulating ear regeneration (9). This and other studies illustrate how DL approaches can facilitate the molecular identification of GRNs and increase the power of traditional genetic and genomic studies for biomedical research.

## Predicting the effect of genetic variation using DL models

The high prediction accuracy of DL models can also be leveraged without understanding the learned sequence rules. When DL models are trained to predict a readout such as ATAC-seq accessibility, the high prediction accuracy holds for similar sequences, including genetic variants within a population or across related species. This makes DL models ideal for the identification of causal genetic variants and has the potential to identify genes and alleles underlying the evolution of complex phenotypes such as mammalian brain size. This extremely challenging problem was tackled by **Irene Kaplow** (Andreas Pfenning laboratory; Carnegie Mellon University) by training a DL model on ATAC-seq data from several well-characterized mammalian brains (10). Her model, TACIT, allowed the identification of several motor cortex enhancers that are associated with the evolution of brain size relative to body size. This work paves the way for using deep learning to identify enhancers and candidate genes involved in complex traits that are subject to evolutionary selection.

Species- and population-level variation was also the subject of DL analysis by **Michael Wilson** (Hospital for Sick Children, Toronto) who applied the BPNet model to mouse liver TFs and showed how it could predict TF binding profiles in other mammalian species. In addition, his laboratory applied this model to interpret variations related to disease-causing alleles involved in blood coagulation and lipid regulation.

Such use of DL models is poised to influence and complement the current genetic approaches used to map and understand the impact of *cis*-regulatory sequence variation. Excellent examples were the talks from Drs Brem, Vierbuchen, Wunderlich, Fay, and Wittkopp. Using a mouse fibroblast senescence model, **Rachel Brem**’s laboratory at UC Berkeley used a classic F1 hybrid approach to identify *cis*-regulatory changes between two mouse species that explains their differential response to irradiation. Their analysis highlighted the TF USF2, which may play a role in senescence decision-making. Similarly, **Thomas Vierbuchen** (Memorial Sloan Kettering Cancer Center) described the use of mouse hybrid cells to link *cis*-regulatory variation with TF binding and enhancer function in the context of mouse embryonic and brain development. **Zeba Wunderlich** (Boston University) studied distinct populations and hybrids of *Drosophila melanogaster* to understand how population-level variation impacts the innate immune response to infection. Her laboratory found that *trans*-acting alleles dominate the response to a Gram-negative infection, while *cis*-acting effects dominate in a Gram-positive infection. A similar population variation approach was used by the laboratory of **Justin Fay** (University of Rochester) to identify protein-based variation linked to thermal tolerance in different species of yeast. They identified numerous differences in protein stability and also an important role of the hybrid cellular environment. **Patricia Wittkopp** (University of Michigan) described her laboratory’s classical genetic approaches to empirically test the assumption that *trans*-acting variants are more pleiotropic than *cis*-acting variants in *Saccharomyces cerevisiae*. By comparing the impact of *cis*- and *trans*-acting mutations on fitness and gene expression, their highly quantitative assays revealed differences in the effects of these two classes of mutations that support the hypothesis that *trans*-regulatory mutations are more pleiotropic than *cis* (11).

The influence of genetic variation can also be studied at the organismal level. **Nicolas Rohner** (Stowers Institute) described work in his laboratory using Mexican cavefish, which independently underwent metabolic adaptation to the cave environment multiple times. His team combined ‘omics datasets from livers of surface and cave morphs to identify putative *cis*-regulatory changes that alter target genes and pathways directly involved in cave adaptation (12). Such a dissection of *cis*-regulatory evolution was also explored by **Phillip Davidson** (Armin Moczek laboratory; Indiana University Bloomington) using dung beetles. These beetles have sexually dimorphic horn development, and in some species, males develop horns in response to nutritional cues. Davidson explored the *cis*-regulatory basis of this developmental plasticity using ‘omics approaches and identified enhancers that may be responsible for nutritional and sex-responsive differential development. In these studies, identification of relevant *cis*-regulatory changes relied on current molecular biological tools including ATAC-seq. It is intriguing to consider how DL approaches may complement this objective in the future.

Using DL models to obtain insights into the mechanisms of gene regulation should be a welcome addition to the current purely experimental approaches. For example, **Evgeny Kvon** (UC Irvine) used chromatin conformation capture technology to map enhancer-promoter interactions for thousands of validated mouse enhancers (13). They concluded that most enhancer-promoter loops are tissue specific and are significantly stronger when enhancers are active. Similarly, **Tathagata Biswas** (Nicolas Rohner laboratory; Stowers Institute) shared his work looking at global chromosomal architecture. These studies make inferences about critical 3D genome interactions that may differ between populations of cavefish. Since DL models can be trained to predict Hi-C data from sequence, these are areas that could benefit from an integrative approach using both DL and targeted experiments.

Several talks at the meeting leveraged the interplay between evolutionary changes and mechanistic insights into gene regulation. By taking evolutionary changes as a starting point, they used classical experimental approaches to uncover specific functions of enhancers, insulators, histone proteins, and TFs, sometimes at the level of a single locus. For instance, **Mark Rebeiz** (University of Pittsburgh) described elegant experiments dissecting evolutionary transformations at the *ebony* locus, where silencers have been systematically reshaped to impact pigmentation in specific *Drosophila* species. Likewise, **Nicolas Gompel** (Ludwig-Maximilians University, Munich) used the *Drosophila* pigmentation system to revisit the notion of enhancer modularity at the *yellow* locus. Through analysis of wing spot pigmentation in a number of species, his laboratory showed how regulatory regions of this gene exhibit multifunctionality and partial redundancy that evolved over time. **Dimple Notani** (National Centre for Biological Sciences, Bangalore) discussed her laboratory’s studies on estrogen-driven gene regulation, where clusters of enhancers appear to act in a cooperative fashion to drive gene expression. Some elements are prebound to the estrogen receptor prior to signaling. Others are induced and appear to require “driver” enhancers for activity. Notably, these elements are not functionally distinguishable based on previously measured chromatin properties.

Exploring evolutionary variation at the protein level, talks by Pravrutha Raman (Harmit Malik laboratory; Fred Hutchinson Cancer Center), David Arnosti (Michigan State University), and Pinar Onal (Shelby Blythe laboratory; Northwestern University) focused on particular TFs and their evolution. **Pravrutha Raman**’s work examines the variability in histone proteins over evolutionary time. She found that ancestral histone variants H2A.X and H2A.Z are found to have fused to form a composite gene, H2A.V in many Diptera. Interestingly, some Drosophila have duplicated the H2A.V variant, with the duplicates expressed in males, indicating that evolutionary innovations in histone proteins may drive biological novelties. **David Arnosti**’s talk about evolution of the C-terminal Binding Protein also investigated how this core component of the transcriptional apparatus has evolved in eukaryotes (14). Deep phylogenetic analysis demonstrated that the intrinsically disordered C terminus bears a surprising level of conservation of short linear motifs, dating back to its earliest last common bilaterian ancestor. Evolution of the Bicoid TF was discussed by **Pinar Onal**, who specifically focused on the role of the Bicoid DNA-binding domain. In testing ancestral forms of this protein in the Drosophila embryo, she was able to replay the evolutionary history of this domain as Bicoid duplicated and evolved (15).

**Figure 1:**
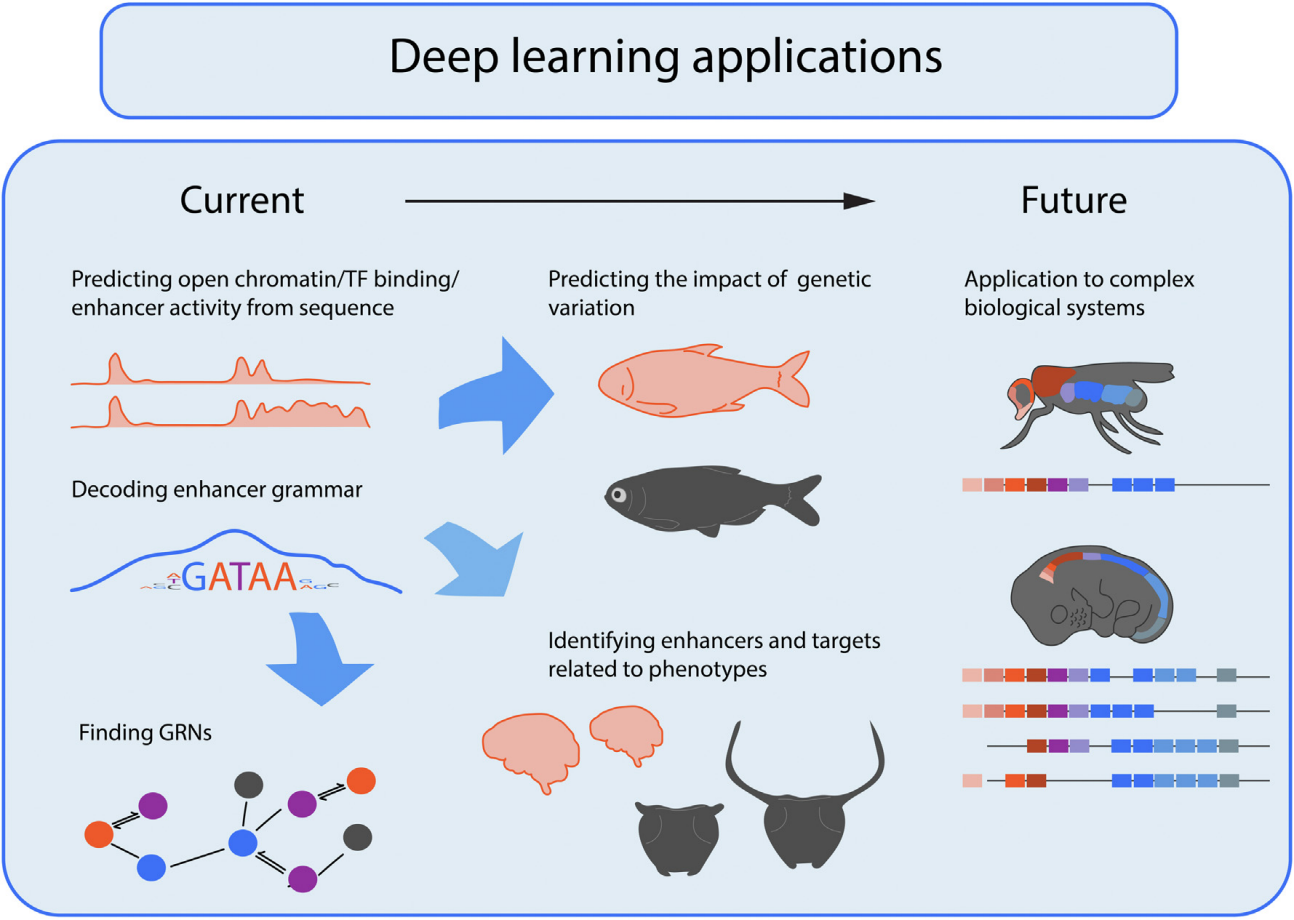
**Applications of deep learning to studying the complexity of gene regulation.** Current deep learning models enable predictions of open chromatin sites, transcription factor (TF) binding, or enhancer activity data from DNA sequence, which can then be interpreted to uncover enhancer grammar and key players of a gene regulatory network (GRN). The prediction capacity is excellent for identifying the impact of genetic variation at a species and population level, such as the difference between cave and surface fish morphs. Altogether, deep learning models are poised to help the identification of enhancers and gene targets involved in evolving traits, *e.g.*, brain size or the phenotypic plasticity of dung beetles in response to nutritional cues. Understanding the activity of complex, highly context-dependent biological systems, such as HOX genes, may represent a future frontier for deep learning.

These focused individual studies demonstrate that learning general rules for gene regulation using DL models should inspire, but not replace, the focus on specific biological problems. Like individual works of art, biological systems need to be considered in their own right. Many aspects by which they operate represent highly specialized solutions to specific organismal challenges. As a result, highly complex combinations of molecular components, such as the HOX gene cluster, are often unique and may only be represented once in a genome (Fig. 1). Thus, while DL innovations are ripe for application to the fields of gene regulation, development, and evolution, they do not replace the unique perspective that individual studies bring.

## Perspectives

The meeting brought together experimental and computational biologists, whose goal is to uncover how gene expression is regulated in the context of evolution. In particular, there was a focus on how DL models can impact the study of gene regulation, development, and evolution (Fig. 1). DL models can be interpreted to identify complex sequence rules that underlie TF binding, enhancer function, open chromatin regions, global chromosomal architecture, and key players in a GRN. These rules can then be tested and explored further using targeted experiments. DL models can also be exploited to make accurate predictions about genetic variation, which together with experimental approaches can help uncover enhancers and target genes involved in specific biological processes and complex phenotypes. Thus, DL models have the potential to become important tools among experimentalists, thereby accelerating unique insights into biological systems.

## Conflict of interest

The authors declare that they have no conflicts of interest with the contents of the article.
